# Ten simple rules for effective presentation slides

**DOI:** 10.1371/journal.pcbi.1009554

**Published:** 2021-12-02

**Authors:** Kristen M. Naegle

**Affiliations:** Biomedical Engineering and the Center for Public Health Genomics, University of Virginia, Charlottesville, Virginia, United States of America

## Introduction

The “presentation slide” is the building block of all academic presentations, whether they are journal clubs, thesis committee meetings, short conference talks, or hour-long seminars. A slide is a single page projected on a screen, usually built on the premise of a title, body, and figures or tables and includes both what is shown and what is spoken about that slide. Multiple slides are strung together to tell the larger story of the presentation. While there have been excellent 10 simple rules on giving entire presentations [1,2], there was an absence in the fine details of how to design a slide for optimal effect—such as the design elements that allow slides to convey meaningful information, to keep the audience engaged and informed, and to deliver the information intended and in the time frame allowed. As all research presentations seek to teach, effective slide design borrows from the same principles as effective teaching, including the consideration of cognitive processing your audience is relying on to organize, process, and retain information. This is written for anyone who needs to prepare slides from any length scale and for most purposes of conveying research to broad audiences. The rules are broken into 3 primary areas. Rules 1 to 5 are about optimizing the scope of each slide. Rules 6 to 8 are about principles around designing elements of the slide. Rules 9 to 10 are about preparing for your presentation, with the slides as the central focus of that preparation.

### Rule 1: Include only one idea per slide

**Fig 1:**
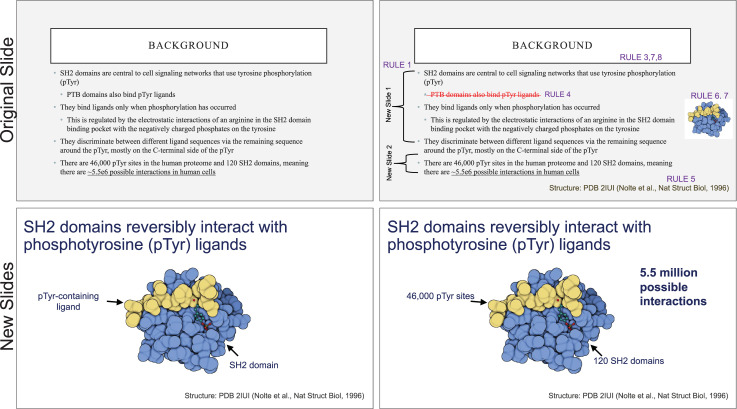
Example of implementing Rules 1–8 to improve a traditional, text-heavy slide. Top left: A background slide that describes the background material on a project from my lab. The slide was created using a PowerPoint Design Template, which had to be modified to increase default text sizes for this figure (i.e., the default text sizes are even worse than shown here). Bottom row: The 2 new slides that break up the content into 2 explicit ideas about the background, using a central graphic. In the first slide, the graphic is an explicit example of the SH2 domain of PI3-kinase interacting with a phosphorylation site (Y754) on the PDGFR to describe the important details of what an SH2 domain and phosphotyrosine ligand are and how they interact. I use that same graphic in the second slide to generalize all binding events and include redundant text to drive home the central message (a lot of possible interactions might occur in the human proteome, more than we can currently measure). Top right highlights which rules were used to move from the original slide to the new slide. Specific changes as highlighted by Rule 7 include increasing contrast by changing the background color, increasing font size, changing to sans serif fonts, and removing all capital text and underlining (using bold to draw attention). PDGFR, platelet-derived growth factor receptor.

Each slide should have one central objective to deliver—the main idea or question [3–5]. Often, this means breaking complex ideas down into manageable pieces (see Fig 1, where “background” information has been split into 2 key concepts). In another example, if you are presenting a complex computational approach in a large flow diagram, introduce it in smaller units, building it up until you finish with the entire diagram. The progressive buildup of complex information means that audiences are prepared to understand the whole picture, once you have dedicated time to each of the parts. You can accomplish the buildup of components in several ways—for example, using presentation software to cover/uncover information. Personally, I choose to create separate slides for each piece of information content I introduce—where the final slide has the entire diagram, and I use cropping or a cover on duplicated slides that come before to hide what I’m not yet ready to include. I use this method in order to ensure that each slide in my deck truly presents one specific idea (the new content) and the amount of the new information on that slide can be described in 1 minute (Rule 2), but it comes with the trade-off—a change to the format of one of the slides in the series often means changes to all slides.

### Rule 2: Spend only 1 minute per slide

When you present your slide in the talk, it should take 1 minute or less to discuss. This rule is really helpful for planning purposes—a 20-minute presentation should have somewhere around 20 slides. Also, frequently giving your audience new information to feast on helps keep them engaged. During practice, if you find yourself spending more than a minute on a slide, there’s too much for that one slide—it’s time to break up the content into multiple slides or even remove information that is not wholly central to the story you are trying to tell. Reduce, reduce, reduce, until you get to a single message, clearly described, which takes less than 1 minute to present.

### Rule 3: Make use of your heading

When each slide conveys only one message, use the heading of that slide to write exactly the message you are trying to deliver. Instead of titling the slide “Results,” try “CTNND1 is central to metastasis” or “False-positive rates are highly sample specific.” Use this landmark signpost to ensure that all the content on that slide is related exactly to the heading and only the heading. Think of the slide heading as the introductory or concluding sentence of a paragraph and the slide content the rest of the paragraph that supports the main point of the paragraph. An audience member should be able to follow along with you in the “paragraph” and come to the same conclusion sentence as your header at the end of the slide.

### Rule 4: Include only essential points

While you are speaking, audience members’ eyes and minds will be wandering over your slide. If you have a comment, detail, or figure on a slide, have a plan to explicitly identify and talk about it. If you don’t think it’s important enough to spend time on, then don’t have it on your slide. This is especially important when faculty are present. I often tell students that thesis committee members are like cats: If you put a shiny bauble in front of them, they’ll go after it. Be sure to only put the shiny baubles on slides that you want them to focus on. Putting together a thesis meeting for only faculty is really an exercise in herding cats (if you have cats, you know this is no easy feat). Clear and concise slide design will go a long way in helping you corral those easily distracted faculty members.

### Rule 5: Give credit, where credit is due

An exception to Rule 4 is to include proper citations or references to work on your slide. When adding citations, names of other researchers, or other types of credit, use a consistent style and method for adding this information to your slides. Your audience will then be able to easily partition this information from the other content. A common mistake people make is to think “I’ll add that reference later,” but I highly recommend you put the proper reference on the slide at the time you make it, before you forget where it came from. Finally, in certain kinds of presentations, credits can make it clear who did the work. For the faculty members heading labs, it is an effective way to connect your audience with the personnel in the lab who did the work, which is a great career booster for that person. For graduate students, it is an effective way to delineate your contribution to the work, especially in meetings where the goal is to establish your credentials for meeting the rigors of a PhD checkpoint.

### Rule 6: Use graphics effectively

As a rule, you should almost never have slides that only contain text. Build your slides around good visualizations. It is a visual presentation after all, and as they say, a picture is worth a thousand words. However, on the flip side, don’t muddy the point of the slide by putting too many complex graphics on a single slide. A multipanel figure that you might include in a manuscript should often be broken into 1 panel per slide (see Rule 1). One way to ensure that you use the graphics effectively is to make a point to introduce the figure and its elements to the audience verbally, especially for data figures. For example, you might say the following: “This graph here shows the measured false-positive rate for an experiment and each point is a replicate of the experiment, the graph demonstrates …” If you have put too much on one slide to present in 1 minute (see Rule 2), then the complexity or number of the visualizations is too much for just one slide.

### Rule 7: Design to avoid cognitive overload

The type of slide elements, the number of them, and how you present them all impact the ability for the audience to intake, organize, and remember the content. For example, a frequent mistake in slide design is to include full sentences, but reading and verbal processing use the same cognitive channels—therefore, an audience member can either read the slide, listen to you, or do some part of both (each poorly), as a result of cognitive overload [4]. The visual channel is separate, allowing images/videos to be processed with auditory information without cognitive overload [6] (Rule 6). As presentations are an exercise in listening, and not reading, do what you can to optimize the ability of the audience to listen. Use words sparingly as “guide posts” to you and the audience about major points of the slide. In fact, you can add short text fragments, redundant with the verbal component of the presentation, which has been shown to improve retention [7] (see Fig 1 for an example of redundant text that avoids cognitive overload). Be careful in the selection of a slide template to minimize accidentally adding elements that the audience must process, but are unimportant. David JP Phillips argues (and effectively demonstrates in his TEDx talk [5]) that the human brain can easily interpret 6 elements and more than that requires a 500% increase in human cognition load—so keep the total number of elements on the slide to 6 or less. Finally, in addition to the use of short text, white space, and the effective use of graphics/images, you can improve ease of cognitive processing further by considering color choices and font type and size. Here are a few suggestions for improving the experience for your audience, highlighting the importance of these elements for some specific groups:

- Use high contrast colors and simple backgrounds with low to no color—for persons with dyslexia or visual impairment.
- Use sans serif fonts and large font sizes (including figure legends), avoid italics, underlining (use bold font instead for emphasis), and all capital letters—for persons with dyslexia or visual impairment [8].
- Use color combinations and palettes that can be understood by those with different forms of color blindness [9]. There are excellent tools available to identify colors to use and ways to simulate your presentation or figures as they might be seen by a person with color blindness (easily found by a web search).
- In this increasing world of virtual presentation tools, consider practicing your talk with a closed captioning system capture your words. Use this to identify how to improve your speaking pace, volume, and annunciation to improve understanding by all members of your audience, but especially those with a hearing impairment.

### Rule 8: Design the slide so that a distracted person gets the main takeaway

It is very difficult to stay focused on a presentation, especially if it is long or if it is part of a longer series of talks at a conference. Audience members may get distracted by an important email, or they may start dreaming of lunch. So, it’s important to look at your slide and ask “If they heard nothing I said, will they understand the key concept of this slide?” The other rules are set up to help with this, including clarity of the single point of the slide (Rule 1), titling it with a major conclusion (Rule 3), and the use of figures (Rule 6) and short text redundant to your verbal description (Rule 7). However, with each slide, step back and ask whether its main conclusion is conveyed, even if someone didn’t hear your accompanying dialog. Importantly, ask if the information on the slide is at the right level of abstraction. For example, do you have too many details about the experiment, which hides the conclusion of the experiment (i.e., breaking Rule 1)? If you are worried about not having enough details, keep a slide at the end of your slide deck (after your conclusions and acknowledgments) with the more detailed information that you can refer to during a question and answer period.

### Rule 9: Iteratively improve slide design through practice

Well-designed slides that follow the first 8 rules are intended to help you deliver the message you intend and in the amount of time you intend to deliver it in. The best way to ensure that you nailed slide design for your presentation is to practice, typically a lot. The most important aspects of practicing a new presentation, with an eye toward slide design, are the following 2 key points: (1) practice to ensure that you hit, each time through, the most important points (for example, the text guide posts you left yourself and the title of the slide); and (2) practice to ensure that as you conclude the end of one slide, it leads directly to the next slide. Slide transitions, what you say as you end one slide and begin the next, are important to keeping the flow of the “story.” Practice is when I discover that the order of my presentation is poor or that I left myself too few guideposts to remember what was coming next. Additionally, during practice, the most frequent things I have to improve relate to Rule 2 (the slide takes too long to present, usually because I broke Rule 1, and I’m delivering too much information for one slide), Rule 4 (I have a nonessential detail on the slide), and Rule 5 (I forgot to give a key reference). The very best type of practice is in front of an audience (for example, your lab or peers), where, with fresh perspectives, they can help you identify places for improving slide content, design, and connections across the entirety of your talk.

### Rule 10: Design to mitigate the impact of technical disasters

The real presentation almost never goes as we planned in our heads or during our practice. Maybe the speaker before you went over time and now you need to adjust. Maybe the computer the organizer is having you use won’t show your video. Maybe your internet is poor on the day you are giving a virtual presentation at a conference. Technical problems are routinely part of the practice of sharing your work through presentations. Hence, you can design your slides to limit the impact certain kinds of technical disasters create and also prepare alternate approaches. Here are just a few examples of the preparation you can do that will take you a long way toward avoiding a complete fiasco:

- Save your presentation as a PDF—if the version of Keynote or PowerPoint on a host computer cause issues, you still have a functional copy that has a higher guarantee of compatibility.
- In using videos, create a backup slide with screen shots of key results. For example, if I have a video of cell migration, I’ll be sure to have a copy of the start and end of the video, in case the video doesn’t play. Even if the video worked, you can pause on this backup slide and take the time to highlight the key results in words if someone could not see or understand the video.
- Avoid animations, such as figures or text that flash/fly-in/etc. Surveys suggest that no one likes movement in presentations [3,4]. There is likely a cognitive underpinning to the almost universal distaste of pointless animations that relates to the idea proposed by Kosslyn and colleagues that animations are salient perceptual units that captures direct attention [4]. Although perceptual salience can be used to draw attention to and improve retention of specific points, if you use this approach for unnecessary/unimportant things (like animation of your bullet point text, fly-ins of figures, etc.), then you will distract your audience from the important content. Finally, animations cause additional processing burdens for people with visual impairments [10] and create opportunities for technical disasters if the software on the host system is not compatible with your planned animation.

## Conclusions

These rules are just a start in creating more engaging presentations that increase audience retention of your material. However, there are wonderful resources on continuing on the journey of becoming an amazing public speaker, which includes understanding the psychology and neuroscience behind human perception and learning. For example, as highlighted in Rule 7, David JP Phillips has a wonderful TEDx talk on the subject [5], and “PowerPoint presentation flaws and failures: A psychological analysis,” by Kosslyn and colleagues is deeply detailed about a number of aspects of human cognition and presentation style [4]. There are many books on the topic, including the popular “Presentation Zen” by Garr Reynolds [11]. Finally, although briefly touched on here, the visualization of data is an entire topic of its own that is worth perfecting for both written and oral presentations of work, with fantastic resources like Edward Tufte’s “The Visual Display of Quantitative Information” [12] or the article “Visualization of Biomedical Data” by O’Donoghue and colleagues [13].
